# Transcriptional Regulation of a Chitinase Gene by 20-Hydroxyecdysone and Starvation in the Oriental Fruit Fly, *Bactrocera dorsalis*

**DOI:** 10.3390/ijms141020048

**Published:** 2013-10-09

**Authors:** Wen-Jia Yang, Kang-Kang Xu, Rui-Ying Zhang, Wei Dou, Jin-Jun Wang

**Affiliations:** Key Laboratory of Entomology and Pest Control Engineering, College of Plant Protection, Southwest University, Chongqing 400716, China; E-Mails: yangwenjia10@126.com (W.-J.Y.); kkxu1988@163.com (K.-K.X.); zhangruiying1993@163.com (R.-Y.Z.); anwdou@gmail.com (W.D.)

**Keywords:** *Bactrocera dorsalis*, chitinase, expression pattern, 20-hydroxyecdysone, starvation

## Abstract

Insect chitinases are hydrolytic enzymes that are required for the degradation of glycosidic bonds of chitin. In this study, we identified and characterized a full-length cDNA of the chitinase gene (*BdCht2*) in the oriental fruit fly, *Bactrocera dorsalis*. The cDNA contains an open reading frame (ORF) of 1449 bp that encodes 483 amino acid residues and 126- and 296-bp non-coding regions at the 5′- and 3′-ends, respectively. The *BdCht2* genome has four exons and three introns. The predicted molecular mass of the deduced *BdCht2* is approximately 54.3 kDa, with an isoelectric point of 5.97. The 977 bp 5′ flanking region was identified and the transcription factor binding sites were predicted. Bioinformatic analyses showed that the deduced amino acid sequence of BdCht2 had 34%–66% identity to that of chitinases identified in other insect species. Quantitative real-time PCR (qPCR) analyses indicated that *BdCht2* was mainly expressed during the larval-pupal and pupal-adult transitions. The tissue-specific expression showed that the highest expression was in the integument, followed by the fat body and other tissues. Moreover, the expression of *BdCht2* was upregulated significantly upon 20-hydroxyecdysone (20E) at different dose injections after 8 h compared to that of the control. Starvation also increased the expression of *BdCht2* in the third-instar larvae and was suppressed again by re-feeding the insects. These results suggest that *BdCht2* plays an important role in the molting process of *B. dorsalis* larvae and can be regulated by 20E.

## 1. Introduction

Chitin is found in fungi, nematodes and arthropods and plays key roles in maintaining morphology and protecting organisms against external attacks [1,2]. In insects, chitin is a vital component of the cuticles of the epidermis and trachea, as well as the peritrophic matrix (PM) in the midgut lumen [3,4]. During the molting process, some chitin in the old cuticle and PM are degraded and replaced by the newly synthesized chitin [5]. Chitin synthesis is catalyzed by chitin synthases (*CHSs*), which are cataloged into two classes: *CHS1* and *CHS2*. In contrast, chitin degradation is hydrolyzed by the chitinase, which catalyzes the random hydrolysis of *N*-acetyl-β-d-glucosamine β-1,4-glycosidic linkages in chitin and chitodextrins. Insect chitinases belong to the family 18 glycosyl hydrolases, based on the conservation of amino acid sequences and many conserved motifs [6].

Insect chitinases are crucial enzymes responsible for the degradation of the chitin in the cuticle and presumably in the PM during molting [7,8]. To date, it is known that there are rather large and diverse groups of chitinase genes identified in several insect species. For instance, the model genetic organism, *Drosophila melanogaster*, has 22 chitinase and chitinase-like genes. *Anopheles gambiae* has 17 genes encoding chitinase, and *Tribolium castaneum*, a member of the order of Coleoptera, has 16 chitinase family genes [9]. Based on phylogenetic analysis of the catalytic domains, insect chitinase and chitinase-like proteins are classified into eight groups [10]. Multiple chitinase family genes may have various functions during insect growth and development. Based on the technology of RNA interference (RNAi) to ascertain the function of different chitinases during the development of *T. castaneum* [7] and *Spodoptera exigua* [11], it is suggested that a part of the chitinases are essential for cuticle turnover, regulating abdominal contraction and wing expansion. In addition, chitinases may be involved in other physiological processes, such as immune defense [12] and disease control [13].

Chitinase activity in insects is at least in part under hormonal regulation, its activity being regulated by hormones, such as juvenile hormone (JH) and 20-hydroxyecdysone (20E) [14,15]. The influence of the hormones on the expression of chitinase genes has been evaluated in some lepidopteran insects [16,17]. 20E has been shown to stimulate the expression of these chitinase genes, but some of them could be suppressed by the simultaneous application of JH [18].

During insect larval development, a restricted supply of nutrients is critical for metamorphosis. In lepidopteran insects, starvation induces supernumerary molts associated with a high level of hemolymph JH titers [19]. However, in *Psacothea hilaris*, starvation of the fourth instar larvae can lead to precocious metamorphosis exceeding a threshold weight with formatting of unusually small, but morphological normal adults [20]. A previous study also found that the *OnCht* expression level can be decreased by feeding in *Ostrinia nubilalis*, suggesting that this chitinase gene may play important roles in the program of molting [21]. Therefore, it is urgent to clarify the relation between chitinase expression and starvation during the insect molting process.

The polyphagous tephritid fruit fly, *Bactrocera dorsalis*, is an insect pest in the tropical and subtropical areas, damaging more than 250 plant species, including numerous fruits and vegetables [22,23]. At present, it is one of the most destructive pests, mainly because of its extreme ability to evolve resistance to many classes of insecticides [24,25]. There is an urgent need to develop new pest control strategies by targeting vital genes. In recent years, it has been documented that chitinases are essential for insect growth and development, and since chitin is not present in vertebrates, it has been considered as a potential target for insect pest management.

In this study, the cloning of the cDNA and genomic DNA sequences of a chitinase gene from *B. dorsalis* (*BdCht2*), as well as the expression patterns in different developmental stages and different tissues were described. The changes in the *BdCht2* expression patterns after the treatment of 20E and starvation were also investigated. This study may provide some insights for further investigation about the chitin-degrading mechanism in the oriental fruit fly and other insects.

## 2. Results

### 2.1. Sequence Analysis of *BdCht2* cDNA

**Figure 1:**
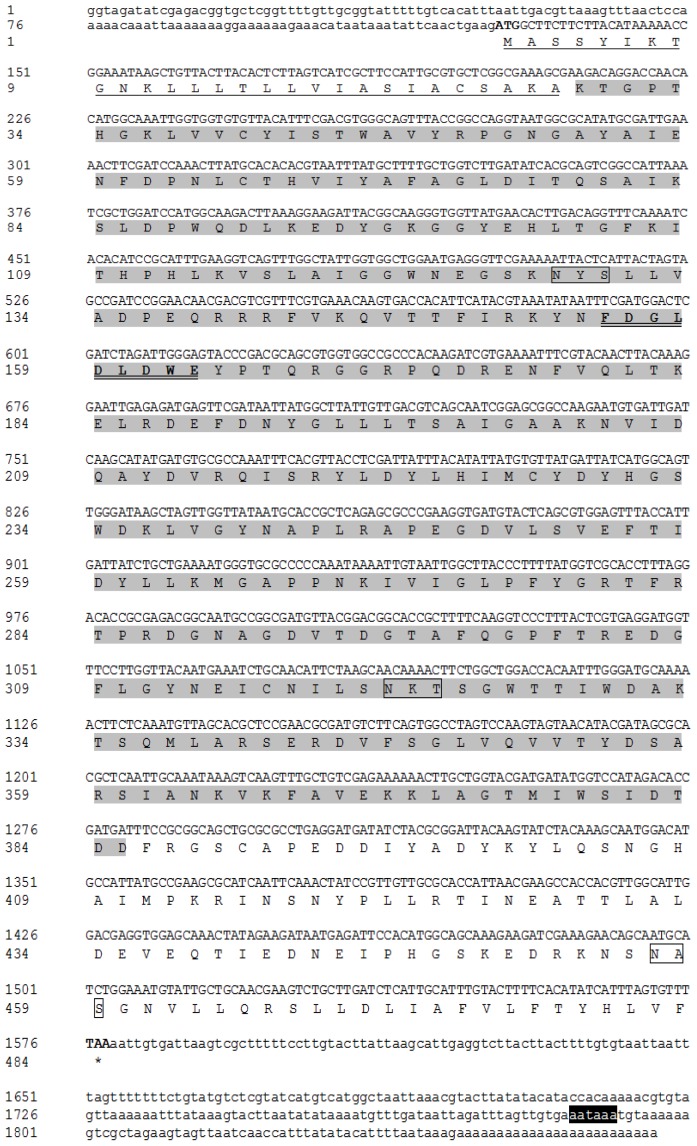
Nucleotide and deduced amino acid sequence of *BdCht2* cDNA in *Bactrocera dorsalis*. The start codon is indicated with bold, and the stop codon is indicated with both bold and an asterisk. The polyadenylation signal (AATAAA) is in white with a black background. The predicted signal peptide is underlined. The conserved family 18 chitinase signature sequence is double underlined. Potential *N*-glycosylation sites are boxed. The position of the putative signal catalytic domain is shaded.

The full-length cDNA sequence of *BdCht2* (GenBank accession number: JN100105) is 1871 bp with an open reading frame (ORF) of 1449 bp, which encodes a protein of 483 amino acids with a predicted molecular mass of approximately 54.3 kDa and an isoelectric point of 5.97. The cDNA includes a 5′-untranslated region (UTR) located 126 bp upstream of the start codon (ATG) and a 3′-UTR of 296 bp that ends in a poly-A tail. The polyadenylation signal (AATAAA) was detected 55 bp upstream from the poly-A tail. The deduced protein BdCht2 seems to be a secretion protein, as a 28-amino acid signal peptide with a cleavage site (Ala 28) in the amino terminal region is present. The *BdCht2* has three potential *N*-glycosylation sites at positions 128, 321 and 457 in the *N*-terminal extracellular domain (Figure 1).

**Figure 2:**
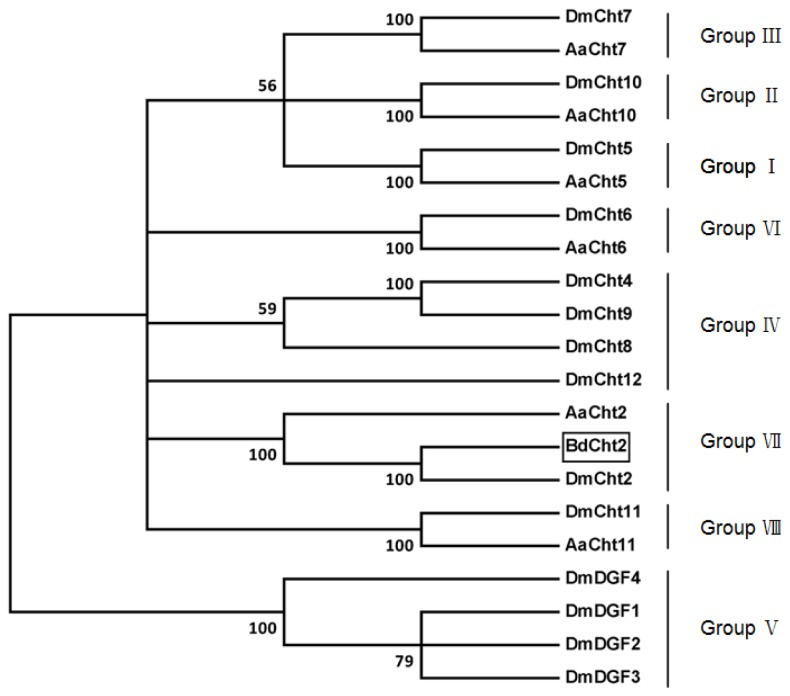
Phylogenetic analysis of chitinase and chitinase-like proteins from three insect species based on catalytic domain sequences. A phylogenetic tree was constructed with MEGA (Molecular Evolutionary Genetics Analysis) 5.04 using the neighbor-joining method. Bootstrap values in percent from 1000 replications are shown. Sequences (GenBank accession numbers) used were: *Aedes aegypti AaCht2* (EAT35472.1), *AaCht5* (EAT45796.1), *AaCht6* (EAT35358.1), *AaCht7* (EAT43684.1), *AaCht10* (EAT35577.1), *AaCht11* (EAT38324.1); *Drosophila melanogaster DmCht2* (NP_477298.2), *DmCht4* (NP_524962.2), *DmCht5* (NP_650314.1), *DmCht6* (NP_572598.2), *DmCht7* (NP_647768.3), *DmCht8* (NP_611542.2), *DmCht9* (NP_611543.3), *DmCht10* (NP_001036422.1), *DmCht11* (NP_572361.1), *DmCht12* (NP_726022.1), *DmDGF1* (AAC99417.1), *DmDGF2* (AAC99418.1), *DmDGF3* (AAC99419.1) and *DmDGF4* (AAC99420.1).

Like the other chitinase 2, *BdCht2* was predicted to contain two domains, including a signal peptide and a single catalytic domain. The glycosylhydrolase_18 conserved domain (FDGLDLDWE) was found in BdCht2. However, no chitin binding domain (CBD) was found at the *C*-terminus. Multiple protein alignments showed that BdCht2 protein had homology to the known and predicted chitinase homologues in other insect species. Identities of BdCht2 with chitinases from *D. melanogaster*, *Chironomus tentans*, *Ostrinia nubilalis* and *Nasonia vitripennis* were 66%, 53%, 36% and 34%, respectively. According to the phylogenetic tree, chitinases were clearly classified into eight separate groups (I–VIII) and BdCht2 belonged to Group VII chitinase, which had only one representative chitinase gene in a variety of insect species (Figure 2).

### 2.2. Genomic DNA Structure and 5′ Flanking Region of *BdCht2*

**Figure 3:**
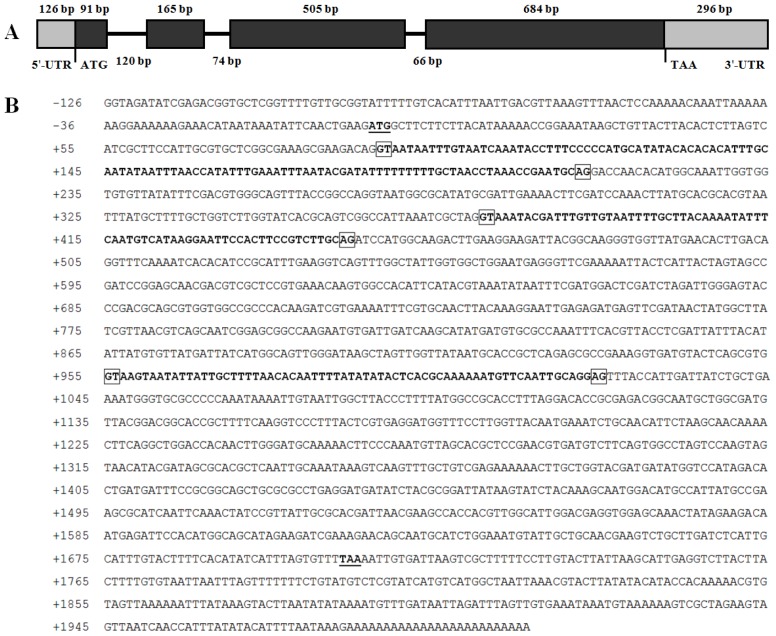
Genomic organization and nucleotide sequence of *Bactrocera dorsalis BdCht2*. (**A**) A schematic representation of the exon and intron organization of *BdCht2. BdCht2* consists of four exons (black boxes) and three introns (intervening line) with a 126 bp 5′ untranslated region (UTR; grey box) and a 296 bp 3′-UTR; (**B**) Genomic DNA sequence of *BdCht2*. The intron sequences are shown in bold. The ATG and TAA are both underlined and indicated with bold. Intron dinucleotide acceptor and donor sites are boxed.

**Figure 4:**
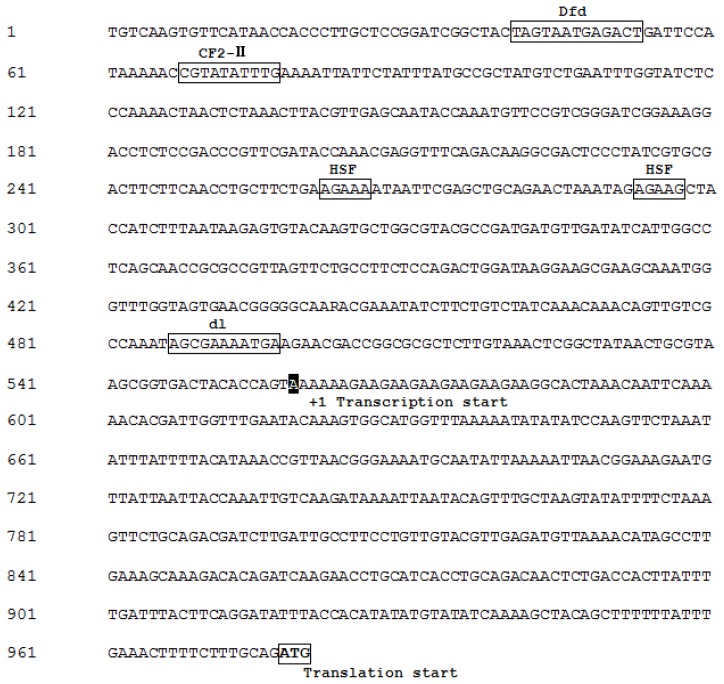
Nucleotide sequence and putative transcription factor binding sites of the 5′-flanking region of *BdCht2*. The translation (ATG) and transcription start sites are boxed and in white with a black background, respectively. The transcription factor binding sites are boxed.

The structure of the gene was determined by comparing the genomic sequence with the *BdCht2* cDNA sequence, and the *BdCht2* genomic sequence (KF289944) was in accordance with its cDNA sequence. The *BdCht2* genomic sequence consists of 1709 bp and is comprised of four exons interrupted by three introns. In addition, the consensus GT and AG sequences at the 5′ donor and 3′ acceptor sites are conserved among the three introns (Figure 3). To identify the regulatory sequences involved in *BdCht2* expression, we isolated a 977 bp DNA fragment upstream of the transcription start site ATG. Transcription factor binding sites were predicted using the TFSEARCH (Transcription Factor Search) software. The predicted binding sites for heat shock factors (HSFs) were identified, which played important roles in heat-induced transcriptional activation, suggesting that *BdCht2* was responsive to heat. Predicted binding sites for CF2-II, which is involved in embryogenesis, were found in the 5′ flanking region of *BdCht2*. In addition, predicted binding sites for Dfd, which is required for normal development of the maxillary segment, and dl, which contributes to regulating the development of dorsoventral polarity, were also observed in the 5′ flanking region of *BdCht2* (Figure 4).

### 2.3. Developmental and Tissue-Specific Expression Patterns of *BdCht2*

**Figure 5:**
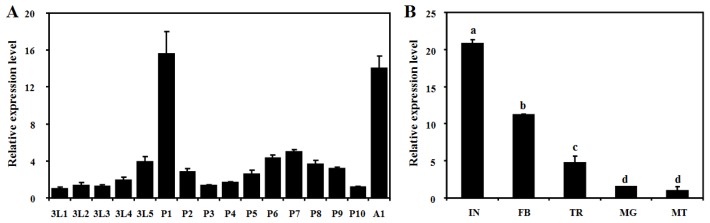
Expression profiles of *BdCht2* as determined by qPCR. (**A**) Expression of *BdCht2* at different developmental stages. 3L1, day-1 third-instar larvae; P1, day-1 pupae; A1, day-1 adults; (**B**) Tissue distribution of *BdCht2* in third-instar larvae. The tissues include integument (IN), fat body (FB), trachea (TR), midgut (MG) and Malpighian tubules (MT). Different letters above each bar indicate significant difference (ANOVA, Duncan’s new multiple range test (DMRT), *p* < 0.05). The error bars represent the means and standard errors of three replicates.

The quantitative real-time PCR (qPCR) method was used to characterize the expression patterns of *BdCht2* during different developmental stages: third-instar larvae, pupae and adults of different ages. The results showed that *BdCht2* mRNA was consistently expressed in all tested stages, suggesting that it has a role throughout these stages. *BdCht2* was mainly expressed during the larval-pupal and pupal-adult transitions, with significantly high expression of the *BdCht2* gene appearing on the day 1 pupae. Low levels of *BdCht2* mRNA were detected early in the third-instar larvae and the other pupal stages (Figure 5A). Additionally, the tissue-specific expression of *BdCht2* analyses showed that it was predominant in the integument, followed by the fat body, then the trachea, midgut and Malpighian tubules (Figure 5B).

### 2.4. Response of the *BdCht2* Expression to the Application of 20E

**Figure 6:**
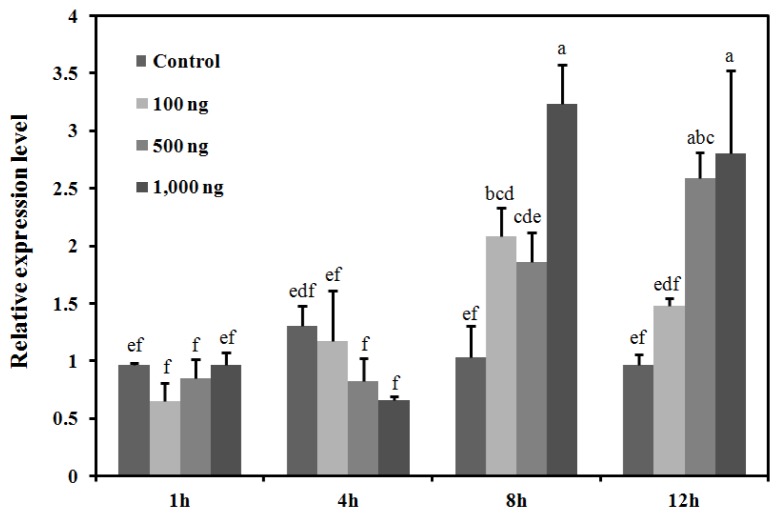
Effect of *in vivo* injection of 20-hydroxyecdysone (20E) on the expression of *BdCht2*. The larvae were collected for qPCR analysis at one, four, eight and 12 h after injection. Control: control insects; 20E (100, 500 or 1000 ng/larva): insects injected with 20E; Different letters above each bar indicate significant difference (ANOVA, DMRT, *p* < 0.05). The error bars represent the means and standard errors of three replicates.

To evaluate the effect of the 20E on *BdCht2* mRNA levels, three different doses of 20E were applied to day 2 third-instar larvae with 0.1% ethanol as the control. The results showed that the *BdCht2* mRNA levels were remarkably induced by 20E, whereas a relatively stable trend in expression of *BdCht2* mRNA was observed in the control group. As for the larvae treated with 1000 ng of 20E, a significant increase in *BdCht2* mRNA was observed at 12 h, and the maximum expression level was at 8 h. By 8 h, treatments with all three doses of 20E had caused a similar induction of *BdCht2* mRNA expression, which increased in a concentration-dependent manner (Figure 6).

### 2.5. Effect of Starvation on Expression of *BdCht2*

**Figure 7:**
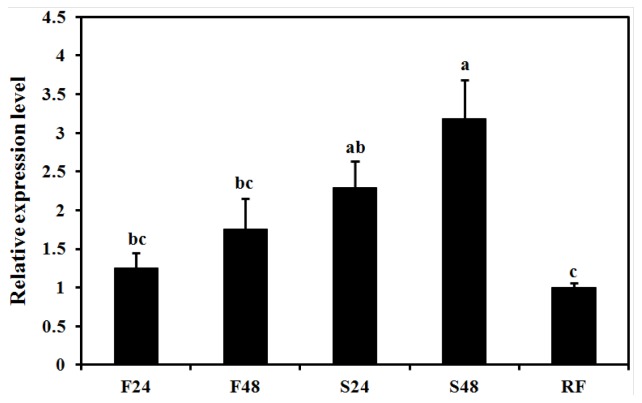
Effect of starvation on the expression of *BdCht2*. The day 2 third-instar larvae were fed or starved for 24 and 48 h before collection. The re-feeding larvae were starved for 24 h first and, then, re-fed for an additional 24 h before collection. F, feeding; S, starvation; RF, re-feeding. Different letters above each bar indicate significant difference (ANOVA, DMRT, *p* < 0.05). The error bars represent the means and standard errors of three replicates.

To determine the effect of feeding on *BdCht2* expression, the day 2 third instar larvae were fed or starved for 24 h and 48 h, then re-fed for 24 h after 24 h of starvation. The results showed that the *BdCht2* expression was significantly upregulated after 48 h starvation compared to the fed insects. However, the expression of *BdCht2* was downregulated when the larvae were re-fed for 24 h after 24 h in starvation (Figure 7). The changes in expression level of *BdCht2* in response to food suggest that it may play an important role in regulating the growth and development of larvae during feeding.

## 3. Discussion

Chitinase is considered as a potential target for insect control, due to its importance in chitin degradation during insect molting [2]. A variety of chitinase and chitinase-like proteins have been reported in different insect species, with emphasis on protein activities and specific functions of individual chitinases and their response to inhibitors [10]. To date, the cDNAs encoding chitinase and chitinase-like proteins have been identified in at least 15 insect species, including: five dipterans, *Glossina morsitans morsitans* [12], *Aedes aegypti*, *A. gambiae*, *D. melanogaster* [26], *Chironomus tentans* [27]; eight lepidopterans, *Spodoptera frugiperda* [8], *S. exigua* [11], *Manduca sexta* [17], *O. nubilalis* [21], *Spodoptera litura* [28], *Choristoneura fumiferana* [29], *Helicoverpa armigera* [30], *Tenebrio molitor* [31]; and two coleopterans, *T. castaneum* [8] and *Apriona germari* [32]. Analyses of these cloned genes will provide us with an opportunity to study the biological functions in different insect species. In this study, we identified and characterized *BdCht2* from the oriental fruit fly, *B. dorsalis*. The *BdCht2* cDNA has a molecular structure consisting of a single peptide, a single catalytic domain and no CBD, which is similar to the domain architecture of Group IV chitinases [10]. The presence of a conserved motif in the catalytic domain that has the consensus, FDGDLDWE motif, suggested that it belonged to the family of 18 glycosyl hydrolases. Based on the typical structure and phylogenetic analyses, *BdCht2* belong to Group VII chitinases.

Chitinases have different expression patterns at different developmental stages depending on specific and distinct functions. In *B. dorsalis*, a higher expression level of *BdCht2* was detected during the larval-pupal and pupal-adult transitions, which suggested that *BdCht2* may be involved in the degradation of chitin for insect molting. Interestingly, the chitin synthase 1 of *B. dorsalis* was also mainly expressed during this molting phase, suggesting that chitin degradation occurs almost at the same time as chitin biosynthesis [33]. Previous studies in *Tribolium castaneum* showed that *TcCht2* was expressed at developmental stages from larval to pupal, but not in embryonic stages or adults [7]. In *A. gambiae*, *AgCht2* was expressed at almost all developmental stages, including eggs, four different larval instars, pupae and adults and mainly expressed in pupae and third-instar larvae [34]. Developmental expression of different chitinase genes exhibited substantial differences in expression patterns of individual groups of chitinase proteins. In *S. exigua*, for example, two chitinase genes were found to be increased substantially before each molting and in the prepupae period, as well as in the eclosion stage [11]. Therefore, further investigations are needed for the elucidation of the roles of other chitinases in *B. dorsalis*.

The *BdCht2* transcript was abundant in the integument of third-instar larvae and lower levels in the midgut, fat body, Malpighian tubules and trachea. It was found that *AgCht2* was expressed in the foregut [34], and *TcCht2* was expressed in the carcass [10]. Taken together, these data demonstrated that *Cht2* in different species may have different functions. There are several reports showing that the integument is the primary site of chitinase expression, including *T. castaneum* [8], *M. sexta* [17], *S. litura* [28] and *C. fumiferana* [29]. These integument-specific chitinases may be involved in cuticle chitin degradation and, subsequently, affect the insect development. Moreover, several chitinases were found to be mainly expressed in the gut of many insect species and presumed to responsible for regulating the chitin content of the PM [7,21,31]. Besides, in *G. morsitans*, a fat body-specific chitinase gene was identified, and it may play a role in immune defense against chitin-containing pathogens [12].

It was reported that gut-specific chitinase expression could be regulated by starvation in many insects. Specifically, in blood-feeding insects, such as *A. gambiae* [35] and *Lutzomyia longipalpis* [36], the expression of chitinase genes was upregulated substantially in response to feeding. In *O. nubilalis*, the transcript level of *OnCht* increased significantly through feeding and was downregulated through starvation [21]. These results suggested that chitinases were involved in changing the gut chitin contents during feeding. However, there has been no report on integument-specific chitinase in response to feeding to date. As starvation induces precocious metamorphosis in the larvae, we propose that the expression of *BdCht2* is precisely coordinated to control the degradation of chitin during the molting process. In the present study, the expression of *BdCht2* was increased by the starvation treatment, but decreased again by the re-feeding treatment, suggesting that *BdCht2* may play roles in the molting process.

Many chitinase genes in various insects are at least, in part, under hormone regulation, their expressions being regulated by hormones, such as 20E and the JH analog (JHA) [15,16,29]. Notably, for minimum endogenous interference, the ideal stage in which the endogenous hormone titers are low should be selected to investigate the precise effect of the treatment of hormone. 20E titers have been shown to be correlated with the abundance of transcripts for the chitinase gene in *T. molitor* during metamorphosis. Application of 20E in pupae alone or in combination with JHA resulted in an induction of transcripts for the *TmChit5* gene [16]. Here, we also found that the *BdCht2* mRNA level could be induced by 20E 8 h after treatment. However, in *B. mori*, upregulation of chitinase expression by 20E can be interfered with simultaneously by JHA treatment [15]. No effect of the treatment of JH alone on chitinase expression was found in *M. sexta* [17]. Besides, the 20E agonist, tebufenozide (RH5992), induced expression of *Cfchitinase* in the integument during the early stages of the sixth instar and caused larvae to undergo an incomplete molting into an extra larval stage [29]. In insects, the expression of more than one chitinase gene was regulated by 20E, and these effects were indirect and might be due to some transcription factors [37].

RNAi has been successfully used to study the gene function of chitinases in diverse groups of insects. In this regard, reports on RNAi-mediated knockdown of various chitinase genes resulting in lethal phenotype are encouraging in coleopteran [7]. Moreover, knockdown of two chitinase genes (*SeChi* and *SeChi-h*) in *S. exigua* resulted in different degrees of survival at the pupation and eclosion stages [11]. Although we performed RNAi for *BdCht2* in *B. dorsalis* by injection of dsRNA into the third-instar larvae, no phenotypic abnormalities were observed, due to a limited RNAi response in the larvae (data not shown), consistent with observations in *A. gambiae* [34]. Similarly, the knockdown efficiency of dsRNA for *TcCht2* was low; there was also no observable adverse effect in the treated larvae [7]. However, whether and how chitinase 2 contributes to insect development is inconclusive and requires further investigation.

## 4. Experimental Section

### 4.1. Test Insect

The stock colony of the oriental fruit fly, *B. dorsalis*, was reared in the laboratory at 27 ± 1 °C and 70% ± 5% relative humidity on a 14 h Light:10 h Dark photoperiod using an artificial diet, as described previously [38]. Insects at different developmental stages were collected, immediately frozen in liquid nitrogen and stored at −80 °C.

### 4.2. Cloning of the Full-Length *BdCht2* cDNA

Total RNA was isolated from the third-instar larvae using TRIzol Reagent (Invitrogen, Carlsbad, CA, USA) following the manufacturer’s instructions. An additional DNase digestion was performed using DNase (TaKaRa, Dalian, China). First-strand cDNA was synthesized by PrimeScript^™^ RT enzyme (TaKaRa, Dalian, China) using random hexamer primers and oligo dT as reverse transcript primers and was used as a template for PCR.

Based on the high-throughput transcriptome sequencing of *B. dorsalis* [39], we identified a chitinase homologous sequence. This sequence was 1310 bp in length, but lacked a coding sequence at the 3′ end. To obtain the full-length cDNA sequence of *BdCht2*, 3′-RACE (rapid amplification of cDNA ends) first strand cDNA were synthesized and a PCR system was constructed according to the instructions of the SMARTer^TM^ RACE cDNA Amplification Kit (Clontech, Palo Alto, CA, USA). Specific primers (Table 1) were used for 3′-RACE, which were synthesized based on the known fragments. The 3′-RACE used 2 μL of 3′-ready-cDNA with Universal Primer Mix (UPM, Clontech, Palo Alto, CA, USA) and *BdCht2*-3GSP1 primer. Then, the nested PCR was carried out with Nested Universal Primer (NUP, Clontech, Palo Alto, CA, USA) and *BdCht2*-3GSP2 primer. The PCR conditions were as follows: 3 min at 95 °C, followed by 34 cycles of 30 s at 95 °C, 30 s at 60 °C and 90 s at 72 °C, then 10 min at 72 °C. After that, to verify the full-length of cDNA, *BdCht2*-F and *BdCht2*-R (Table 1) were designed to amplify the open reading frame (ORF) of *BdCht2*. The PCR products were separated by 1.5% agarose gel electrophoresis and stained with GoodView^™^ (SBS Genetech, Beijing, China).

### 4.3. Cloning of the Genomic Sequences and 5′-Flanking Region of *BdCht2*

Genomic DNA was extracted from adults using an EasyPure Genomic DNA Extraction Kit (TransGen, Beijing, China) according to the manufacturer’s instructions. One pair of gene-specific primers (*BdCht2*-F/*BdCht2*-R) was used to produce the genomic DNA sequence of *BdCht2* with *B. dorsalis* genomic DNA as a template. Exons and introns were identified by comparing and analyzing the cDNA and genomic DNA sequences. The 5′-flanking region of *BdCht2* was obtained by a Genome Walking Kit (TaKaRa, Dalian, China), and PCR reactions were carried out as per the manufacturer’s instruction.

### 4.4. Cloning and Sequencing

A band of the expected size was excised, and the fragment was purified using the Wizard^®^ SV Gel and PCR Clean-Up System (Promega, Madison, WI, USA) and, then, cloned into pGEM^®^-T Easy vector (Promega, Madison, WI, USA). Ligation reactions were used for transformation of DH5α competent cells (Transgen, Beijing, China). Several recombinant clones were identified by PCR with the primers used before and further sequenced in both directions with an ABI (Applied Biosystems, Foster City, CA, USA) Model 3100 automated sequencer (Life Technologies, Shanghai, China).

### 4.5. Bioinformatic Analysis and Phylogenetic Tree Construction

Sequence similarity and analysis for conserved domains were performed using the BLAST (Basic Local Alignment Search Tool) program (http://blast.ncbi.nlm.nih.gov/Blast.cgi). The ORF of *BdCht2* cDNA was identified using the ORF Finder (http://www.ncbi.nlm.nih.gov/gorf/gorf.html). Sequences were edited and multiple sequence alignments were performed with DNAMAN 5.2.2 (Lynnon BioSoft, Quebec, Canada, 2001). The signal peptide was predicted by the SignalP 4.1 Server program (http://www.cbs.dtu.dk/services/SignalP/). Some protein sequence analysis tools used in this study, including theoretic molecular weight, isoelectric point and *N*-glycosylation sites, were obtained from the ExPASy Proteomics website (http://expasy.org/). The online program, SMART (Simple Modular Architecture Research Tool) (http://smart.embl-heidelberg.de/), was used to predict the domain architecture in the amino acid sequences. The gene structure was analyzed by the Spidey program (http://www.ncbi.nlm.nih.gov/spidey/). The transcription factor binding sites in the 5′-flanking region of *BdCht2* were predicted using the TFSEARCH database (http://www.cbrc.jp/research/db/TFSEARCH.html). The phylogenetic tree was constructed by MEGA 5.04 [40] using the neighbor-joining method. Bootstrap values were calculated on 1000 replications.

### 4.6. Developmental and Tissue-Specific Expression of *BdCht2*

To investigate the expression of *BdCht2* in different developmental stages and tissues, the insects from newly-molted third-instar larvae to adults were collected, and five selected tissues, including the integument, trachea, midgut, Malpighian tubules and fat body, were dissected from the third-instar larvae. Total RNA extraction, DNase treatment and cDNA synthesis were performed as described in Section 4.2. Gene-specific qPCR primers were shown in Table 1. The qPCR was performed on an ABI 7500 Real-Time PCR System (Applied Biosystems, Foster City, CA, USA) using the following thermo-cycler conditions: 95 °C for 2 min, followed by 40 cycles of 95 °C for 15 s, 60 °C for 30 s and 72 °C for 30 s. Finally, a melting curve analysis from 60 °C to 95 °C was applied to all reactions to ensure the consistency and specificity of the amplified product. The PCR amplifications were performed in 20-μL reaction systems containing 1 μL of template cDNA, 10 μL iQ^™^ SYBR^®^ Green Supermix (Bio-Rad, Hercules, CA, USA) and 0.2 mM each of the primers. The relative expression analysis for qPCR was performed by using α-Tubulin gene (GU269902) as an internal reference based on our previous evaluations [41]. The ΔΔ*Ct* method was used to analyze the relative differences in the expression levels [42]. Three biological replicates, each with two technical replications were used for qPCR analysis. Data were statistically analyzed using one-way analysis of variance (ANOVA), and the means were separated by Duncan’s new multiple range test (DMRT) for significance (*p* < 0.05) by using SPSS 16.0 for windows (SPSS Inc., Chicago, IL, USA, 2008).

### 4.7. 20E Treatment

The 20-hydroxyecdysone (20E; Sigma, St. Louis, MO, USA) was dissolved in 95% ethanol to make a storage concentration of 10 μg/μL. The storage solutions were then diluted to 1 μg/μL with distilled water. The day 2 third-instar larvae were used for 20E treatment. The treatment method following the micro-topical application technique was performed as previously reported [33]. For the 20E treatment group, the insects were injected with three different doses of 20E (100, 500 and 1000 ng/larva). For the control group, the larvae were only injected with an equivalent volume of 0.1% ethanol. All the treated larvae were reared as described above. In 1, 4, 8 and 12 h after the topical application of 20E, the insects were collected and the *BdCht2* mRNA level was determined as described above.

### 4.8. Gene Expression Patterns in Feeding and Starvation Treatment

In the starvation experiments, the day 2 third-instar larvae were used and divided into three groups each with 30 larvae. The first group was used as the control and maintained on the artificial diet until sample collection. The second group was starved for 24 h or 48 h before sample collection. The third group was starved for 24 h and then re-fed for the next 24 h. All of the treated larvae were collected to determine the *BdCht2* mRNA level as described above. Three biological replications, each with two technical replications, were used in this analysis.

## 5. Conclusions

In conclusion, this study has demonstrated the molecular characterization and expression patterns of *BdCht2* in *B. dorsalis*. The putative BdCht2 belongs to Group VII chitinases. Developmental and tissue-specific analysis of *BdCht2* suggests that *BdCht2* may be involved in the degradation of chitin for the integument during insect molting. In addition, we also investigated *BdCht2* mRNA expression after 20E injection and found that *BdCht2* expression was upregulated. Furthermore, *BdCht2* expression was induced by starvation and repressed by feeding. However, RNAi for *BdCht2* has no observable adverse effect in the treated insects. Further studies to identify the mechanisms responsible for RNAi may help us to further understand the molecular mechanism of the chitinase gene in *B. dorsalis*.

## Figures and Tables

**Table 1:** PCR primers used in this study.

Name sequence	Primer sequence (5′–3′)	Usage
*BdCht2*-3GSP1	GAAGATTACGGCAAGGGTGGT	3′-RACE
*BdCht2*-3GSP2	CCATAGACACCGATGATTTCCG	

*BdCht2*-F	CTGAAGATGGCTTCTTCTTACATAA	cDNA Full-length confirmation
*BdCht2*-R	CAATTTTAAAACACTAAATGATATG	

*BdCht2*-SP1	TCCGGATCGGCTACTAGTAATGAG	Cloning 5′-flanking regions
*BdCht2*-SP2	GTCAAGTGTTCATAACCACCCTTGC	

Q-*BdCht2*-F	ATCACACATCCGCATTTGAA	Quantitative real-time PCR
Q-*BdCht2*-R	CGTCGGGTACTCCCAATCTA	
Q-α-Tubulin-F	CGCATTCATGGTTGATAACG	
Q-α-Tubulin-R	GGGCACCAAGTTAGTCTGGA	
